# High Frequency of Bipolar Disorder Comorbidity in Medical Inpatients

**Published:** 2019-01

**Authors:** Atefeh Ghanbari Jolfaei, Samaneh Ataei, Raoofeh Ghayoomi, Amir Shabani

**Affiliations:** 1 Minimally Invasive Surgery Research Center, Department of Psychiatry, Iran University of Medical Sciences, Tehran, Iran.; 2 Department of Community Psychiatry, School of Behavioral Sciences and Mental Health, Iran University of Medical Sciences, Tehran, Iran.; 3 Mental Health Research Center, Bipolar Disorders Research Group, Iran University of Medical Sciences, Tehran, Iran.

**Keywords:** *Bipolar Mood Disorder*, *Comorbidity*, *Frequency*, *General Hospital*, *Medical Disease*

## Abstract

**Objective:** Bipolar disorder is a severe, disabling, and recurring disorder.

Some studies have shown that the frequency of bipolar disorder in patients with medical diseases is higher than healthy controls. The aim of this study was to investigate the frequency of bipolar disorders in medically ill patients hospitalized in Iranian general hospitals.

**Method** **:** In this cross sectional study, 697 inpatients (342 men, 49.1%) from different wards of 3 general hospitals, with the mean age of 39.3+-10, were enrolled in the study using nonprobability sampling. Demographic questionnaire, Mood Disorder Questionnaire (MDQ) and Bipolar Spectrum Diagnostic Scale (BSDS) were used. Inclusion criteria were as follow: informed consent, age 18-65 years, ability to speak Persian, and having at least middle school education.

**Results: **The frequency of bipolar disorder was 12.1% and 20.8% based on BSDS and MDQ, respectively. The results of both tests were positive in 7.9% of hospitalized patients. The frequency of bipolar mood disorder was significantly higher in single patients and in those with comorbidity of alcohol and substance use disorders.

**Conclusion: **Considering the high frequency of bipolar mood disorders in hospitalized medically ill patients and its probable effects on compliance and prognosis, early screening, diagnosis, and treatment of bipolar mood disorders is important in these patients.

Epidemiological studies have a high priority in determining national and international policies and are inspiring and directional in clinical and basic investigations and researches. The role of epidemiological studies is important in defining disorders, revealing pathology, describing risk factors, and creating a vision about different therapeutic aspects (1). 

Bipolar disorder is known as manic depression in the third edition of Diagnostic and Statistical Manual of Mental Disorders (DSM), and although some studies consider continuity between bipolar disorder and depressive disorders, the clinicians usually split mood disorders into 2 divisions: (1) bipolar disorders, (2) depressive disorders (2, 3). 

Bipolar disorder is a serious, disabling, and recurring disorder (4) and despite numerous pharmacological/ biological and psychological tr eatments, it has a high rate of morbidity and mortality (5). Also, it can cause functional and psychological disability (6) and increase the risk of suicide (7). According to the undermining and recurrent nature of bipolar disorder (8), early diagnosis and intervention may reduce its negative consequences. 

The prevalence of bipolar disorder in general population is different among studies. Hardoon et al. (9) stated that these different statistics may be due to various sampling methods, various diagnostic tools, locational diversity, and varied observational periods. Blanco et al. (10) found the 12-month prevalence of bipolar disorder type 1 to be 1/5% to 2/1%, based on DSM- 5 criteria, and found no difference between males and females. 

The aggregate lifetime prevalence of all types of bipolar disorder was reported to be 5% (11). Previous studies showed that bipolar disorder is accompanied by special medical diseases and psychiatric disorders (12) which decrease quality of life (13) and increase morbidity and mortality (14). Despite the increasing number of studies on bipolar disorders in psychiatric outpatient and inpatient settings, our knowledge about the rate of bipolar disorder through medical settings is limited (15). The related epidemiological researches imply that bipolar disorder type 1 is highly comorbid with alcohol dependency (16), substance use disorders (17), and suicide (18). In addition, McElroy and Keck (19) studied medical comorbidities of bipolar disorder and showed that this disorder is concomitant with cardiovascular diseases, diabetic type 2, and metabolic syndrome. In agreement with their study, Gálvez et al. (20) presented that the rate of obesity in bipolar patients is more than the general population. In addition, other studies indicated that bipolar disorder is also associated with other medical disorders, including epilepsy (21), chronic pain (22), liver diseases (23), neurologic diseases, pulmonary diseases (including asthma and chronic obstructive pulmonary disease), endocrine diseases (including hyperthyroidism), kidney diseases, gastrointestinal diseases, and AIDS (24). 

The aim of this study was to investigate the frequency of bipolar disorder in medical patients hospitalized in general hospitals of Iran University of Medical Sciences.

## Materials and Methods

This was a descriptive epidemiological study. The target sample of this study was the medical inpatients of Rasoul-Akram, Firoozabadi, and Firoozgar hospitals. The sample included 706 patients who were selected using nonprobability sampling. Inclusion criteria were as follow: age 18-65 years, ability to speak Persian, and having at least middle school education. However, being mentally retarded, having cognitive disorders (such as dementia and delirium), being admitted to ICU and CCU were determined as exclusion criteria. Written informed consent was obtained from all the patients.

The research instruments were 3 questionnaires, including Demographic Questionnaire, the Persian version of Mood Disorder Questionnaire, and Bipolar Spectrum Diagnostic Scale. 

Mood Disorder Questionnaire (MDQ): This self-reporting scale, which was designed by Hirschfield et al. in 2000, is a useful screening tool for diagnosing bipolar disorder and should be filled by patients. This scale includes 3 parts. The first part includes 13 questions and screens the patients based on the symptoms of mania or hypomania throughout the life time. The second part of the questionnaire determines the existence of current mania and hypomania symptoms. The third part checks the effect of the disorder in overall function. The questionnaire’s overall reliability by means of Cronbach alpha equaled 0.81. The sensitivity and specificity were calculated to be 67% and 76%, respectively. The psychometric properties of the Persian version of this questionnaire were reported by Masaeli et al. (25).

Bipolar Spectrum Diagnostic Scale (BSDS): This questionnaire is a bipolar spectrum screening scale and includes 2 parts. The first part starts with a short story by addressing the patient as a third person and contains 19 sentences, including the subtle symptoms of bipolar disorder. In case the person could relate to a sentence, 1 point is given to him/her. The second part, which include multiple-choice questions, is given to the person upon the appropriate score which ranges from 0 to 6. The maximum acquirable scores is 25. According to Shabani et al. (26) study, the sensitivity and specificity of the Persian Bipolar Spectrum Diagnostic Scale at the cut-off point of 14, were 0.52 and 0.79, respectively.

## Results

A total of 760 patients [342 males (49.1%) and 355 females (50.9%)] enrolled in the study; of them, 9 were excluded because of uncompleted data. The mean age of the participants was 39.3+-10. With respect to marital status, 16.7% were single, 77.3% were married, and 2.4% and 3.6% were divorced and widowed, respectively. Moreover, 273 patients (39.2%) had graduated from elementary school, 270 (38.8%) had high school diploma, and 154 (22.1%) had a university degree. Also, 52.8% of the patients were employed, 3.3% were unemployed, 36.6% were housewives, and 7.3% were retired. 

**Chart 1:**
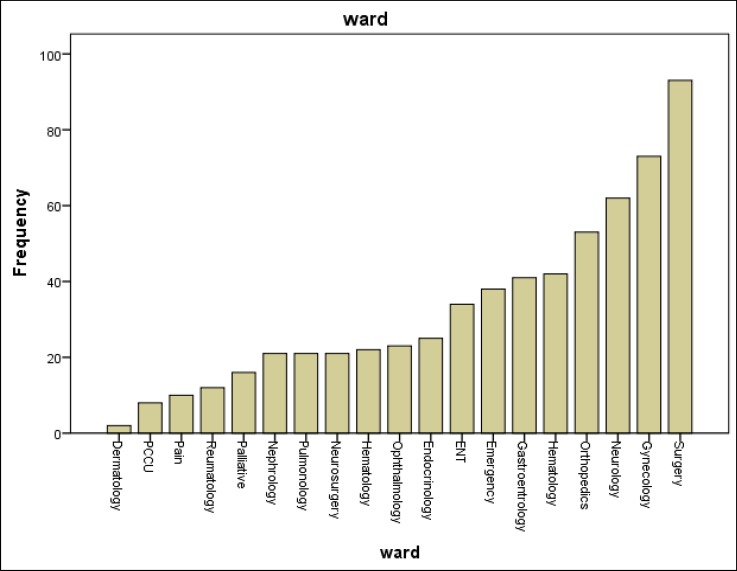
Bipolar Disorder Frequency in Inpatients of Different Wards

According to BSDS questionnaire, the frequency of bipolar disorder was 12.1 in medical inpatients in hospitals of Iran University of Medical Sciences; this frequency was 20.8 based on the MDQ questionnaire, and 7.9% based on both questionnaires. The correlation between the 2 scales (BSDS and MDQ) was 0.407. The results also revealed that the frequency of bipolar disorder based on the BSDS was higher in orthopedic, rheumatology, neurosurgery, and pulmonology wards, which was 27.1%, 20%, 17.8%, and 17.8% respectively; and the least rate of the disorders based on BSDS was in urology, dermatology, and ENT (0%). Furthermore, the highest frequency of bipolar disorders based on MDQ scale was reported in orthopedic (18.6%), pulmonology (17.8%), and rheumatology (10%) wards; however, comorbidity with bipolar disorder was not diagnosed in orology, cardiology, and ENT wards. The chart 1 shows the distribution of enrolled participants from each ward.

According to the results of Table 1, based on BSDS scores single patients (19 %) showed a more significant bipolar disorder rate compared to the married (10%). Also, smokers (25 %) showed a more significant bipolar disorder rate compared to non-smokers (10%). Patients with alcohol use disorders (40%) demonstrated a more significant bipolar disorder rate compared to others (10%). Regarding substance use disorders, it was concluded that patients with substance use disorders (38%) had a more significant bipolar disorder rate comorbidity (11%). MDQ scores were a little different (Table 2).

**Table 1:** The Difference of Bipolar Disorder Frequency throughout Various Variables Based on Bipolar Spectrum Diagnostic Scale

**Variable**	**Level of Difference**	**Chi** **-** **Square Test**	**Significance Level**
Gender	Male = Female	3.281	0.070
Marital statues	Married < Single	8.912	0.030
Cigarette smoking	No use < Consumer	20.723	0.001
Alcohol use disorders	No use < Consumer	23.103	0.001
Substance use disorders	No use < Consumer	13.856	0.001

**Table 2:** The Difference of Bipolar Disorder Frequency throughout Various Variables Based on Mood Disorder Questionnaire

**Variable**	**Level of Difference**	**Significance Level**	**Chi** **-** **Square Test**
Gender	Female < Male	0.027	4.895
Marital status	Married < Single	0.044	8.118
Smoking	No use < Consumer	0.005	7.730
Alcohol use disorders	No use < Consumer	0.008	7.012
Substance use disorders	No use = Consumer	0.373	0.793

## Discussion

The aim of this study was to determine the frequency of bipolar disorder in hospitals of the medical inpatients in Iran University of Medical Sciences. In the BSDS questionnaire, the frequency of bipolar disorder was 12.1%; this rate was 20.8% according to MDQ, and it was 7.9% based on the results of both questionnaires. 

If we consider the results of each questionnaire and those of other studies (11), which have shown that the life time prevalence of bipolar disorder in healthy population is about 5%, it can be implied that the frequency of bipolar disorder in medical inpatients is about 2 to 4 times higher than the general population. In a research by Pouretemad et al. (27) in Iran, the rate of bipolar mood disorder was 0.8%. Stubbs et al. (28) reported that the prevalence of bipolar disorder was 1.9% in primary care clinics. 

In this study, it was found that the prevalence of bipolar disorder in medically ill patients was far more frequent than the general population. This finding agrees with Carney& Jone (24), McElroy & Keck (19), and Galvez et al, (29) who indicated that medical illnesses have a high comorbidity with bipolar disorder. It seems that in addition to the probability of common basic biological and genetic factors, the environmental stressors can also be mentioned as one of the probable reasons of this high rate of comorbidity. 

In studies on neurobiology of bipolar disorders, it was frequently reported that in addition to changes of serotonin, dopamine also increases in patients with mania or hypomania (30). Furthermore, according to Vaessen et al. study (31), the outbreak and increase of dopamine was reported in medical illnesses, so it can be concluded that medical and bipolar disorder may have common neurobiological etiology, and dopamine increases in both states. 

In addition, the other probable factor that can be the determinant of this high comorbidity is the life style of bipolar patients, which exposes them to medical diseases more frequently (32). In this regard, it was demonstrated that bipolar patients are more vulnerable to obesity, type 2 diabetes, and cardiovascular diseases because of unhealthy diet, and lack of exercise and physical activity. (33). Vancampfort et al. (34) also indicated that low physical activity and improper diet of bipolar patients increase the risk of other medical diseases in these patients. 

The other factor that can increase the comorbidity of medical diseases in bipolar disorder patients is impulsivity. Feki et al. (35) showed that the level of impulsivity among bipolar patients in comparison with the general population is higher, the rate of impulsivity was 55%. Thus, in the present study, the orthopedic issues of these patients and higher rate of hospitalization of patients with bipolar disorder in the orthopedic ward may have been the result of impulsivity and reckless behaviors. 

The other issue is the side effects of medication in patients with bipolar disorder. Researches have confirmed that these medications disturb the metabolic process which can lead to medical illnesses (36). 

It was also indicated that the frequency of bipolar disorder is more in medically ill males. This finding agrees with that of Grant et al. (37) that indicated that bipolar disorder type 1 is more frequent in males than in females. On the other hand, some researchers have found that bipolar disorder type 2 and rapid cycling are more common among females (38). These diversities may be due to the use of different measuring tools. Moreover, different types of bipolar disorder were not differentiated in the present study and if this separation had been applied, then different results could have been obtained. 

Also, it was shown that bipolar disorder in single patients were significantly more frequent compared to the married. This finding is in contradiction with some other studies. Blanco et al. (11) and Kim et al. (39) have declared that the prevalence of bipolar disorder is not significantly different between single and married patients. Regarding the difference in the results of the present study (in medical patients), it can be stated that the role of the partner as a protecting factor gains more significance and because single individuals are deprived from this powerful support, they are more susceptible to mental disorders and medical diseases. Besides, in this study, it was found that cigarette smoking and alcohol and drug use disorders are significantly more frequent among the medically ill bipolar patients. The findings of this study agree with several researches, which revealed a connection among these variables (11, 40-41). Some studies have introduced alcohol and drug use disorders as susceptive factors in triggering or worsening bipolar disorder (42). On the other hand, some other studies presented these disorders as the consequence of bipolar disorder. Alcohol and drug use disorders are considered as some type of self-medication for bipolar patients and consequently they can worsen the medical condition and create a type of mal-cycle (22). In addition, alcohol and drug use disorders can affect the hypothalamus-pituitary-adrenal axis and may release dopamine (43), leading to the aggravation of bipolar symptoms.

We suggest that the researchers replicate this study by paying attention to the categorization of types of bipolar disorder and using clinical interview for diagnosis.

## Limitation

Overall, it is suggested to view this study by taking its limits into consideration, including not differentiating between the types of bipolar disorder, specifically types 1 and 2. The other limitation of this study was its reliance on questionnaire and not using clinical interviews. In a study by Carvalho et al. (44) at recommended cut-offs, summary sensitivity for the MDQ and BSDS was 66% and 69%, while specificity was 79% and 86% for both questionnaires, respectively. In fact, both of these tests have the potential of “false positives.” Moreover, considering the sampling method, the participants in this study were not representative of all patients with medical diseases. In addition, the questionnaires were difficult to understand for some of the patients and we tried to provide explanations to them, however, this problem might have affected the accuracy of the results.

## Conclusion

The frequency of bipolar mood disorder in medically ill patients hospitalized in general hospitals is significant, due to the impact of the disorder on the lifestyle, treatment adherence, course and the prognosis, identification and treatment of bipolar mood disorder is important in these patients.
